# Supplementary material for: Characterization of a Coproduct from the Sea Cucumber Cucumaria frondosa and Its Effects on Visceral Adipocyte Size in Male Wistar Rats
Source: Mar Drugs. 2020 Oct 26;18(11):530. doi: 10.3390/md18110530 (PMC7693588; doi:10.3390/md18110530)
Supplement: Supplementary file 1 [file marinedrugs-18-00530-s001.pdf]

marine drugs

Characterization of a Coproduct from the Sea Cucumber *Cucumaria frondosa* and its Effects on Visceral Adipocyte Size in Male Wistar Rats

Alan Ramalho [1,2], Nadine Leblanc [2], Marie-Gil Fortin[3], André Marette [2,4,5], André Tchernof [1,4] and Hélène Jacques [1,2,*]

[1] School of Nutrition, Faculty of Agricultural and Food Sciences, Université Laval, Québec, G1V 0A6, Canada; alan.ramalho.1@ulaval.ca (A.R.); andre.tchernof@criucpq.ulaval.ca (A.T.)
[2] Institute of Nutrition and Functional Foods, Université Laval, Québec, G1V 0A6, Canada; Nadine.Leblanc@fsaa.ulaval.ca (N.L.); Andre.Marette@criucpq.ulaval.ca (A.M.)
[3] Fisheries and Aquaculture Innovation Centre, Merinov, Gaspé, G4X 2V6, Canada; Marie-Gil.Fortin@merinov.ca
[4] Quebec Heart and Lung Institute, Université Laval. Québec, G1V 4G5, Canada
[5] Department of Medicine, Faculty of Medicine, Université Laval, Québec, G1V 0A6, Canada
* Correspondence: Helene.Jacques@fsaa.ulaval.ca

Supplementary Table S1. Amino acid content of sea cucumber coproduct protein (mean ± standard deviation; n= 4 replicates except for tryptophane where n=3) (g/100 g of protein).

	Amino acid	Concentration	Amino Acid Score[2]
Essential	Histidine	1.54 ± 0.13	1.03
	Isoleucine	3.18 ± 0.38	1.06
	Leucine	5.51 ± 0.31	0.93
	Lysine	5.33 ± 0.34	1.18
	Methionine	1.55 ± 0.14	0.97
	Phenylalanine	3.31 ± 0.29	1.58[1]
	Threonine	4.11 ± 0.21	1.79
	Tryptophan	0.57 ± 0.05	0.95
	Valine	3.80 ± 0.38	0.97
Non-essential	Alanine	4.71 ± 0.25	N.A.
	Arginine	4.48 ± 0.29	N.A.
	Aspartic acid	6.90 ± 0.23	N.A.
	Cysteine	0.93 ± 0.15	1.55
	Glutamic acid	10.4 ± 0.5	N.A.
	Glycine	5.06 ± 0.27	N.A.
	Proline	3.48 ± 0.11	N.A.
	Serine	3.53 ± 0.15	N.A.
	Tyrosine	2.69 ± 0.17	N.A.

[1] The amino acid score for phenylalanine is calculated using the sum of the contents of phenylalanine and tyrosine. [2] The amino acid scores were calculated according to the method outlined by the FAO/WHO/UNU using the adult indispensable amino acid requirements as determined by the FAO/WHO/UNU [92]. To calculate the scores, the amino acid content of the coproduct (mg amino acid /g protein) was divided by the requirement pattern of the same amino acid for adults (mg amino acid /g protein) [92]. N.A. not applicable

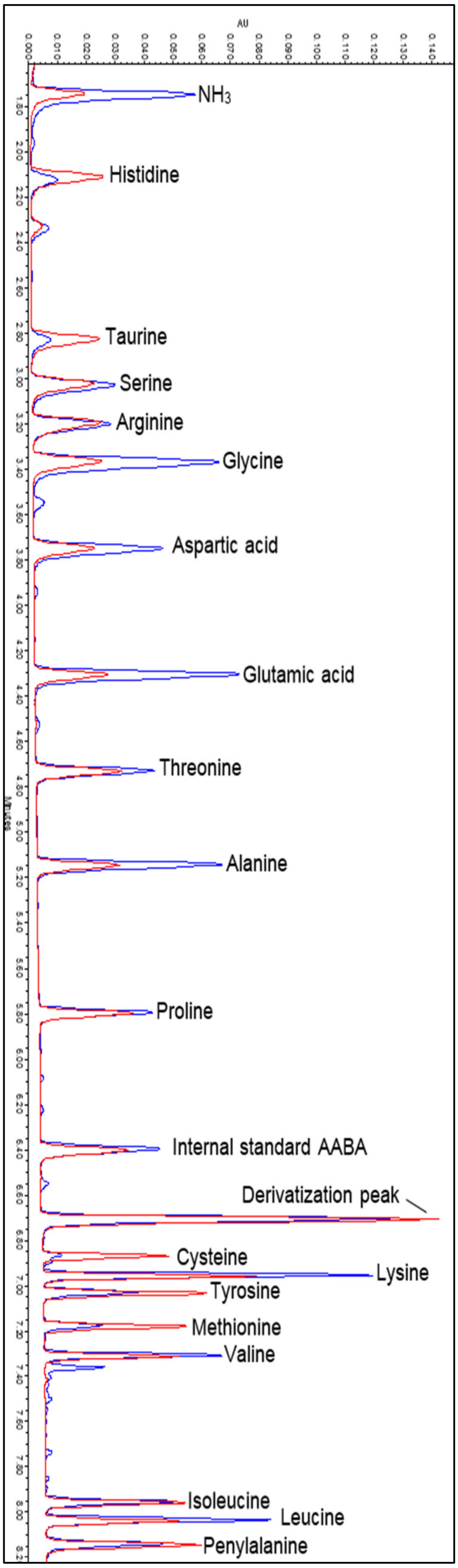

Supplementary Figure S1. Representative chromatogram of amino acids analysis by UPLC-PDA. In red, amino acids standards and in blue, a sample of amino acids after digestion of sea cucumber coproduct.

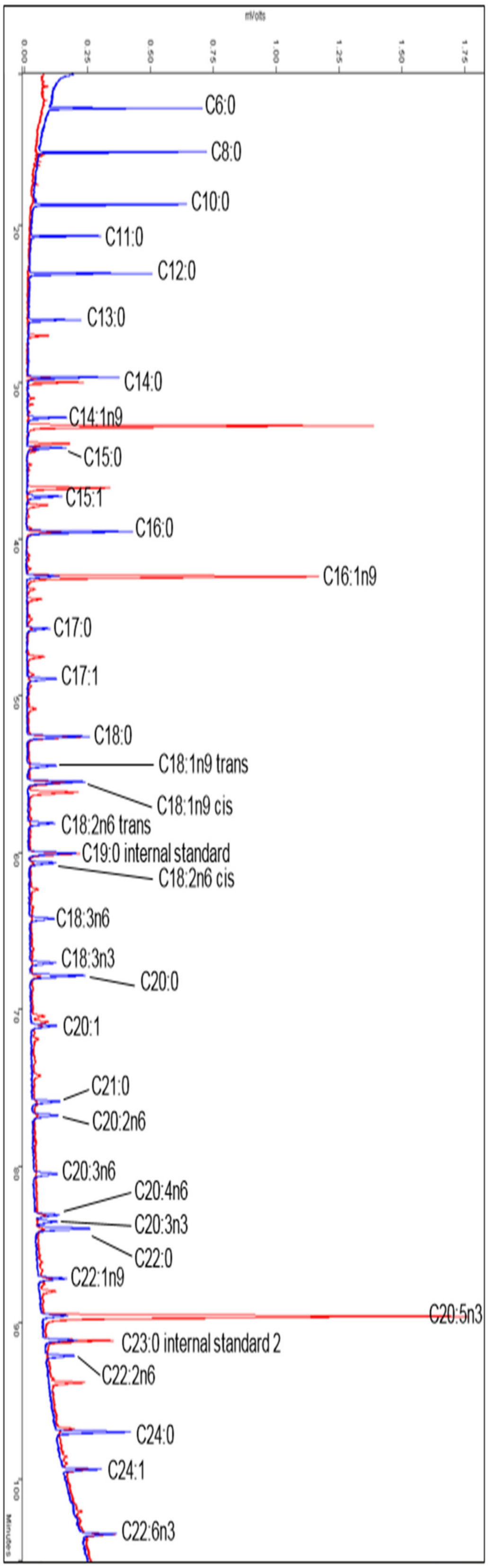

Supplementary Figure S2. Representative chromatogram of fatty acids analysis by GC-FID. In blue, a Supelco 37 Component FAME Mix and in red, a sample of fatty acids from sea cucumber coproduct.

Supplementary Table S2. Complete fatty acid profile of the lipids in the dried sea cucumber coproduct (mg/g lipid).

Fatty acid	**Retention time (min)**	**Replicate 1**	**Replicate 2**	**Replicate 3**
C6:0				
C8:0				
C10:0				
C11:0				
C12:0				
C13:0				
Unidentified 1	26.99	6.75	4.65	7.13
C14:0	29.67	4.91	5.46	4.44
Unidentified 2	29.97	9.5	10.83	9.73
Unidentified 3	32.74	56.21	69.03	58.31
C14:1n9 cis				
Unidentified 4	33.87	8.58	9.26	9.11
C15:0				
C15:1				
Unidentified 5	36.67	17.36	19.11	18.58
Unidentified 6	37.76	5.47	5.61	6.3
C16:0	39.27	8.43	8.38	9.36
C16:1n7 cis	42.07	68.57	73.71	72.69
C17:0				
C17:1				
C18:0	52.49	14.01	13.76	15.14
C18:1n9t				
C18:1n9c	55.52	12.88	13.17	13.5
C18:1n7 cis	56.13	14.21	13.97	15.27
C18:2n6 trans				
C18:2n6 cis	60.23			
C18:3n6				
C18:3n3	66.56			
C20:0	67.86	4.34	3.09	4.4
C20:1	71.05	2.38	2.05	2.73
C21:0				
C20:2n6	76.78	2.07	1.19	2.4
C20:3n6				
C20:4n6	83.12	5.01	4.24	4.9
C20:3n3	83.54			
C22:0	83.77			
C22:1n9	87.15	3.95	3.45	3.96
C20:5n3	89.56	145.37	148.30	155.57
C20:4				
C24:0	96.57			
C24:1	98.89	7.93	7.20	8.19
C22:5n3	101.56			
C22:6n3	103.33	7.48	7.13	7.43

Control 0.5%

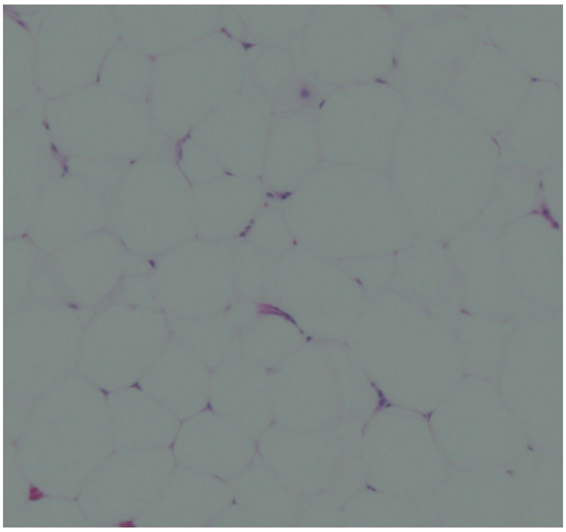

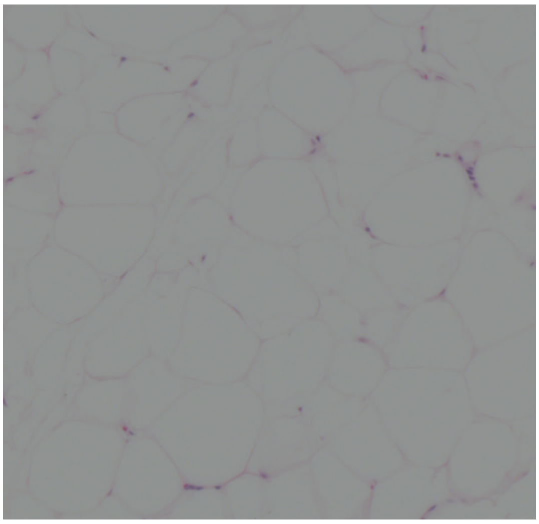

1.5% 2.5%

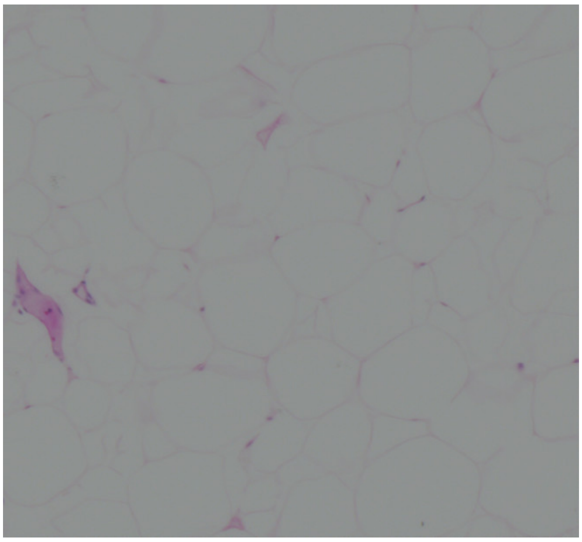

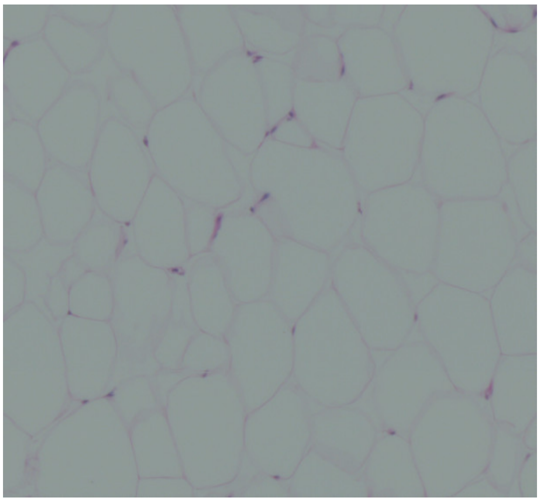

Supplementary Figure S3. Representative histological samples of epididymal adipose tissue subregions fixed in buffered formalin and stained with hematoxylin and eosin from each diet group. The images have been reduced to 35% of their original size while preserving the aspect ratio. The images have not been altered in any other way.

Control 0.5%

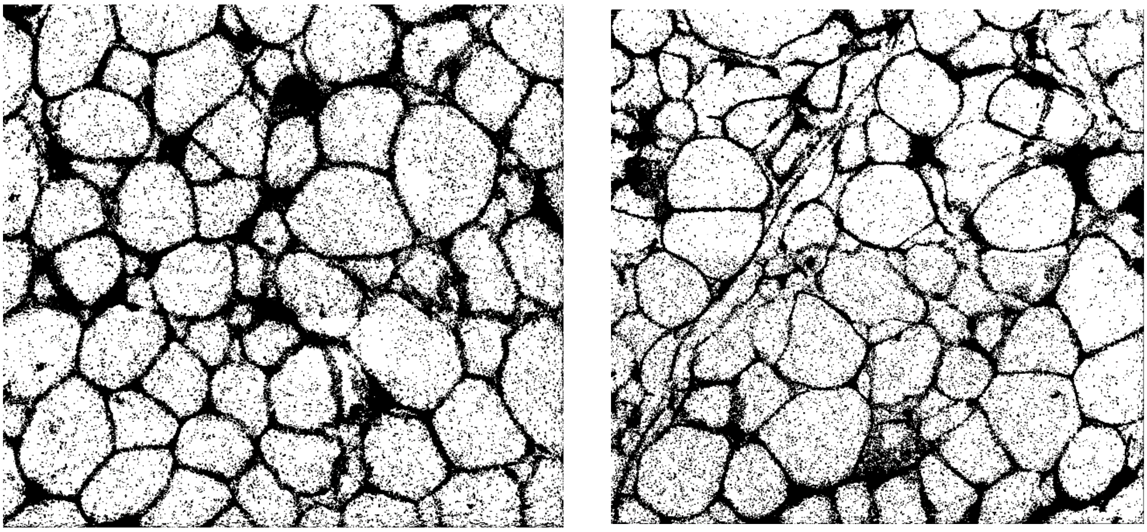

1.5% 2.5%

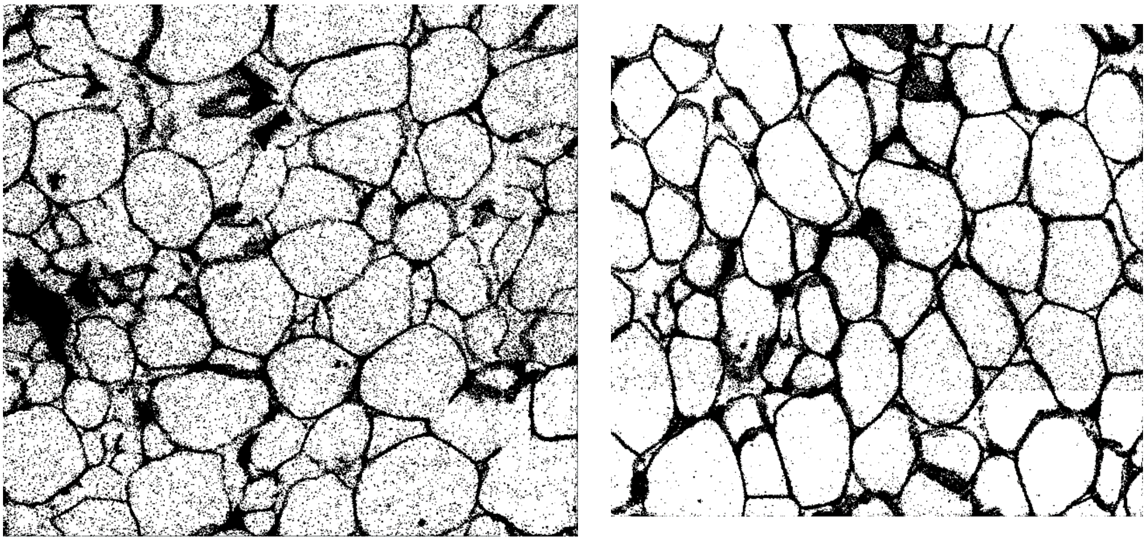

Supplementary Figure S4. Representative histological samples of epididymal adipose tissue subregions after being converted to 8-bit, having the background subtracted, the threshold adjusted, and converted binary. These subregions are the same as in Supplementary Figure S3.

© 2020 by the authors. Licensee MDPI, Basel, Switzerland. This article is an open access article distributed under the terms and conditions of the Creative Commons Attribution (CC BY) license (http://creativecommons.org/licenses/by/4.0/).
